# In vivo efficacy of tobramycin-loaded synthetic calcium phosphate beads in a rabbit model of staphylococcal osteomyelitis

**DOI:** 10.1186/s12941-018-0296-3

**Published:** 2018-12-28

**Authors:** Godday Anebow Lulu, Arunkumar Karunanidhi, Loqman Mohamad Yusof, Yusuf Abba, Fazlin Mohd Fauzi, Fauziah Othman

**Affiliations:** 1 Department of Human Anatomy, Faculty of Medicine and Health Sciences, Universiti Putra Malaysia, 43400 Serdang, Selangor Darul Ehsan Malaysia; 2 Department of Medical Microbiology and Parasitology, Faculty of Medicine and Health Sciences, Universiti Putra Malaysia, 43400 Serdang, Selangor Darul Ehsan Malaysia; 3 Department of Pharmacology and Chemistry, Faculty of Pharmacy, Universiti Teknologi MARA, 42300 Bandar Puncak Alam, Selangor Darul Ehsan Malaysia; 4 Department of Companion Animal Medicine and Surgery, Faculty of Veterinary Medicine, Universiti Putra Malaysia, 43400 Serdang, Selangor Darul Ehsan Malaysia; 5 Department of Veterinary Pathology and Microbiology, Faculty of Veterinary Medicine, Universiti Putra Malaysia, 43400 Serdang, Selangor Darul Ehsan Malaysia; 6 Research Laboratory of Anatomy and Histology, Faculty of Medicine and Health Sciences, Universiti Putra Malaysia, 43400 Serdang, Selangor Darul Ehsan Malaysia

**Keywords:** Tobramycin, Antibacterial activity, Calcium phosphate beads, Osteomyelitis

## Abstract

**Background:**

Osteomyelitis is an acute or chronic inflammatory process of the bone following infection with pyogenic organisms like *Staphylococcus aureus*. Tobramycin (TOB) is a promising aminoglycoside antibiotic used to treat various bacterial infections, including *S. aureus*. The aim of this study was to investigate the efficacy of tobramycin-loaded calcium phosphate beads (CPB) in a rabbit osteomyelitis model.

**Methods:**

Tobramycin (30 mg/mL) was incorporated into CPB by dipping method and the efficacy of TOB-loaded CPB was studied in a rabbit osteomyelitis model. For juxtaposition, CPB with and without TOB were prepared. Twenty-five New Zealand white rabbits were grouped (*n* = 5) as sham (group 1), TOB-loaded CPB without *S. aureus* (group 2), *S. aureus* only (group 3), *S. aureus* + CPB (group 4), and *S. aureus* + TOB-loaded CPB (group 5). Groups infected with *S. aureus* followed by CPB implantation were immediately subjected to surgery at the mid-shaft of the tibia. After 28 days post-surgery, all rabbits were euthanized and the presence or absence of chronic osteomyelitis and the extent of architectural destruction of the bone were assessed by radiology, bacteriology and histological studies.

**Results:**

Tobramycin-loaded CPB group potentially inhibited the growth of *S. aureus* causing 3.2 to 3.4 log_10_ reductions in CFU/g of bone tissue compared to the controls. Untreated groups infected with *S. aureus* showed signs of chronic osteomyelitis with abundant bacterial growth and alterations in bone architecture. The sham group and TOB-loaded CPB group showed no evidence of bacterial growth.

**Conclusions:**

TOB-incorporated into CPB for local bone administration was proven to be more successful in increasing the efficacy of TOB in this rabbit osteomyelitis model and hence could represent a good alternative to other formulations used in the treatment of osteomyelitis.

**Electronic supplementary material:**

The online version of this article (10.1186/s12941-018-0296-3) contains supplementary material, which is available to authorized users.

## Background

Postoperative and post-traumatic osteomyelitis is one of the most serious complication with high rates of associated morbidity and has been categorized as difficult-to-treat infections in the orthopaedic field [1, 2]. Tremendous progress has been made in the past few years aimed at preventing osteomyelitis, especially by using antibiotics with different spectrums of activity against specific pathogens. Operative techniques like debridement and muscle flaps have also been employed to overcome this complication [3–9]. In addition to the systemically administered antibiotics alone, the contemporary prevention strategies using biomaterials like antibiotic-loaded poly(methyl methacrylate) (PMMA) cement spacers and antibiotic-incorporated beads for local administration of antibiotics were comparably more effective in increasing the efficacy of locally-administered antibiotics [10–13].

Treatment of osteomyelitis with local administration of antibiotics using different delivery system, e.g. biodegradable microsphere containing TOB, PMMA beads containing TOB, in combination with systemic antibiotics are some of the choices of treatment [13, 14]. The continued release of TOB from the microsphere and PMMA beads has been demonstrated in the treatment of osteomyelitis [15]. Microspheres containing TOB has also been reported to contain higher local antibiotic concentration, especially in bone and soft tissues compared to systemic levels [14, 15]. While the use of beads have shown considerable effectiveness in the treatment of osteomyelitis, disadvantages such as poor discharge of antibiotics, the inability to incorporate many useful antibiotics because of heat-instability, involvement of secondary surgery in the removal of cements and lack of biodegradable qualities makes them unviable treatment options [16–18]. Given that current treatment options for *staphylococcal* infections have become limited, this creates an urgent need for novel antibiotic delivery systems. Therefore, development of new delivery systems for antibiotics using biomaterials like CPB will not only remove *S. aureus* from infection sites effectively, but can potentially revert the damages inflicted on the bone by subsidizing mineral components, which is advantageous to new bone formation.

A biodegradable drug delivery system would have the merit of avoiding the removal of drug carrier which normally involves a secondary surgery. Furthermore, the discharge rate of antibiotics can be fine-tuned in a biodegradable system, which can be attributed to the properties of the material. The solubilizing property of the carrier does not prevent new bone from growing into the defective site, and the calcium component of the beads serves to replace the bone mineral.

Biodegradable materials like calcium sulfate, collagen, polymers and other microcapsules have been reported to be suitable drug delivery systems in the treatment of osteomyelitis [19]. In our earlier investigation, we have shown that in vitro incorporation of tobramycin and gentamycin with CPB resulted in a slow residual release of the antibiotic from 30 min to 1344 h (8 weeks) with dissolution of calcium phosphate [20]. However, incorporating TOB with CPB has not been tested in vivo in an animal model for the prevention of osteomyelitis. Therefore, the aim of the present study was to investigate the efficacy of TOB incorporated CPB in a rabbit osteomyelitis model.

## Materials and methods

### Preparation of CPB loaded with TOB

Commercially available calcium phosphate beads (JectOS^®^ liquid and powder, Kasios^®^, LʹUnion, France) was prepared according to the methods described previously [20]. Briefly, the beads were prepared by pouring 5 mL of jectos liquid and 3 mL of 1% soluble starch (BDH, United Kingdom) into a sterile bowl. Then 10 g of jectos powder was added and mixed vigorously with spatula until the mixture turned to a smooth homogenous liquid (~ 30 min). Then, using a 1 mL syringe, the liquid paste was filled and slowly extruded on a petri dish to form ~ 0.2 g bead. The prepared beads were allowed to dry overnight and sterilized by gamma irradiation (Malaysia Nuclear Agency) at a measured dose of 25.1 kGy. Tobramycin antibiotic (Sigma Aldrich, St. Louis, MO) was then loaded into CPB by dipping the beads into TOB solution at room temperature (21 °C) and allowing it to stand for 24 h at 4 °C.

### Animals

Twenty-five specific pathogen free adult male New Zealand White rabbits (Sapphire Enterprise, Selangor, Malaysia) weighing between 2.5 and 3 kg were used in this study. The animals were allowed to acclimatize for 3 weeks before surgery in the animal housing facility of Universiti Putra Malaysia (UPM), in a 12:12 light–dark cycle. All experiments were carried out in accordance with the guidelines of the Institutional Animal Welfare Committee; the study protocol was approved by the Animal Ethical Committee with approval number: UPM/IACUC/AUP-R030/2013. Animals were housed in individual cages and were provided with antibiotic free rabbit diet (Perternakan Hong Lee Sdn Bhd, Malaysia) and tap water ad libitum. Rabbits were divided into five groups with four animals in each group and the details of grouping are as follows: group 1—normal control; group 2—untreated group implanted with TOB-loaded CPB; group 3—untreated group inoculated with *S. aureus*; group 4—treated group implanted with CPB and inoculated with *S. aureus* and group 5—treated group implanted with TOB-loaded CPB and inoculated with *S. aureus*.

### Bacterial strain and inoculum preparation

Reference strain of *S. aureus* ATCC 12600 (*S. aureus* strain Xen 29, ATCC Manassas, VA, USA) was used in this study [21]. The strain was grown in tryptic soy broth (TSB, Becton–Dickinson and Company) at 37 °C. Prior to surgery, bacterial inoculum containing ~ 10^7^ CFU/mL in phosphate buffered saline (PBS) was prepared. Aliquot of ~ 5 × 10^7^ CFU/mL were obtained and re-suspended to a final volume concentration of 2 × 10^7^ CFU/mL. Cell suspensions were formulated on the day of surgery and held on ice until implantation. From this suspension, a volume of 100 µL of inoculum was injected into the midshaft of the tibia. After surgery, the bacterial load present in 1 g of bone tissue was serially diluted and plated on TSA. After 28 days post-surgery, the presence of bacteria in bone tissues was evaluated based on the colony forming units (CFU) in 1 g of bone.

### Operative procedure

Osteomyelitis of the tibia was induced by following the procedure described previously [22]. Briefly, the rabbits were weighed and anaesthetized with a combination of 5 mg/kg xylazine (Rompun, Bayer Vital GmbH, Leverkusen, Germany) and 35 mg/kg of ketamine (Ketaset, Zoetis, New Jersey, USA) via subcutaneous injection. Anaesthesia was maintained with free flow inhalation of 1.5% isoflurane (Primal Critical Care Inc., Pennsylvania, USA). Then the rabbits were placed on recumbence and the right knee and hock area was shaved and surgically prepared with chlorhexidine, 70% alcohol and finally with tincture iodine. Prior to surgery, the surgical area was isolated from the rest of the body by placing a sterile surgical drape. A small skin incision of about 3–5 mm was made at the anterior surface of the tibia. The periosteum was sharply incised and bluntly elevated from the midshaft. Using an 18 guage needle, a hole of ~ 0.2 cm was made at the midshaft of the tibia. Subsequently, the midshaft of 12 rabbits were inoculated with 100 µL of *S. aureus* inoculum containing 2 × 10^7^ CFU/mL by microinjection using a sterile pipette tip with an exterior diameter of 0.6 mm directly into the medullary canal. Immediately after challenging with *S. aureus* at the surgical site, a prepared CPB was inserted and placed directly over the hole at the midshaft of 12 rabbits (see Additional file 1: Fig. S1). The fascial layer was then replaced to its initial position and closed immediately with a coated VICRYL^®^ 4-0 suture (Polyglactin 910, Ethicon Inc., New Jersey, USA) to prevent leakage of the inoculum and to secure the CPB in place. The control group (*n *= 4) received plain CPB without TOB. The TOB treated group (*n *= 4) received a total of 30 mg/mL of TOB. Osteomyelitis infection group (*n *= 4) were inoculated with *S. aureus* ATCC 12600 only. Test group (*n *= 4) were implanted with TOB-loaded CPB without bacteria. And the sham group (*n *= 4) was operated but left untreated. After surgery, the surgical sites were closed with stitches and disinfected, and all rabbits received 0.05 mg/kg of buprenorphine (PharmaForce Inc., Ohio, USA) as a post-operative analgesic, subcutaneously. Osteomyelitis of the right tibia was allowed to develop and was confirmed by X-ray after 28 days post-surgery. Animals were monitored daily for 28 consecutive days for locomotion status, food and water intake and signs of localized and systematic infection. Changes in body weight and body temperature were also recorded for their correlation to infection.

### Post mortem and sample acquisition

On 28 days post infection (dpi), rabbits were euthanized with intravenous injection of 75 mg/kg pentobarbital sodium (Dolethal, Vetoquinol S.A., Lure cedex, France). The tibiae of each rabbit were subjected to radiographical, bacteriological, histological and macroscopic analysis. The infected bone and surrounding soft tissues of the tibia were removed. The right tibia was retrieved and stored in 10% buffered formal saline in airtight glass containers before it was processed for histological analysis. Prior to euthanasia, blood samples were drawn for hemogram analysis and erythrocyte sedimentation rate (ESR) evaluation at 0, 7, 14, 21 and 28 dpi. Bone samples were also obtained from experimental rabbits for bacteriological and histological analysis. The tibiae of the experimental rabbit was radiographed at three stages of the study i.e., before surgery, immediately after surgery and 28 dpi in order to monitor the progression of osteomyelitis.

### Microbiological analysis

Two transverse slices of ± 0.5 cm (~ 0.9 g) from the tibia were sawn; the proximal section was used for bacteriological determination, while the distal section was used for histological analysis. The proximal section was homogenized in 20 mL of PBS and the suspension was serially diluted in trypticase soy agar (TSA) plate. After 24 h incubation, the bacterial colony counts were enumerated to represent the CFU/g of bone. The average detection limit was set at 2500 CFU/g of bone and a full-blown infection was characterized by a bacterial load that reached 10% of the initial inoculum dose (> 10^5^ CFU/g). Colonies formed on TSA were further subcultured onto mannitol salt agar for the presumptive detection of *S. aureus* (Oxoid, Malaysia).

### Radiological analysis

Odekerken’s scoring system was used to confirm the signs of osteomyelitis, where a maximum score of four signifies severe osteomyelitis [23]. The specific morphological changes around the infected midshaft of the tibia were graded as follows: 0—no radiologic abnormalities; 1—mild periosteal reaction with mild osteolysis; 2—periosteal reaction and evident osteolysis; 3—periosteal reaction with calcification in the subperiostium, extension of osteolysis to diaphysis and cortical thickening; 4—extensive metaphyseal osteolysis.

### Histopathological analysis

Bone samples were harvested and fixed in 10% buffered formal saline and histologically processed according to a previously described method with minor modifications [24]. Tissue sections were stained with Masson Goldner’s Trichrome and the slides were examined under a light microscope (Olympus, Germany). The disease status was assessed based on the scores of acute intra-medullar inflammation, chronic intra-medullar inflammation, periosteal inflammation and bone necrosis (Table 1). New bone formation was assessed based on the microanatomy scoring system (Table 2).

**Table 1:** Osteomyelitis scoring system-histology (Masson Goldner’s trichrome stain)

Osteomyelitis grade	Morphological changes
0	No histopathological changes
1	Mild periosteal inflammation
2	Severe periosteal inflammation
3	Acute intra-medullar inflammation
4	Chronic intra-medullar inflammation
5	Bone necrosis

**Table 2:** Microanatomy scoring system (Masson Goldner’s trichrome stain)

Microanatomy grade	Microanatomy changes
0	Decrease bone formation Destruction of bone architecture
1	Increase bone formation Retention of bone architecture

### Statistical analysis

Hematological data and the changes in body weight and temperature were analyzed by two way analysis of variance (ANOVA) followed by Tukey’s multiple comparison of groups. The differences in bacterial loads between groups were determined by Mann–Whitney *U*-test for non-parametric two-tailed significance. The radiology, histology and microanatomy data were analyzed by Kruskal–Wallis test followed by Dunnett’s or Tukey’s multiple comparison test. A *p* value of < 0.05 was considered statistically significant and graphical representation of the data was performed using GraphPad Prism^®^ version 6.01 (GraphPad, San Diego, CA, USA).

## Results

### Physical examination

**Fig. 1:**
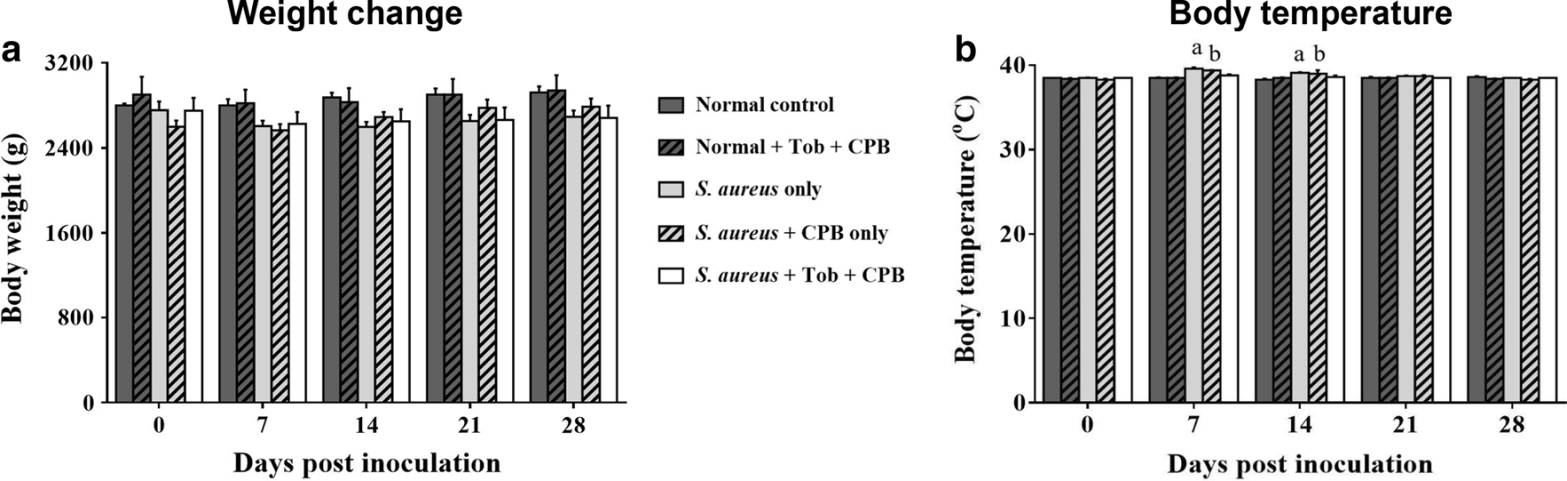
Physiological parameters of experimental rabbits (**a**) body weight change (**b**) body temperature during rabbit follow-up in control groups and bacteria induced implant groups. ^a,b^Means with different superscript are statistically significant once compared to respective day 0. Error bars represent the standard error of the mean. ^a^*p* ≤ 0.001, ^b^*p* ≤ 0.01

All rabbits fully recovered after the surgery and had inappetence on the first day of surgery. There were no significant differences in the body weights of rabbits in all groups until at 28 dpi (Fig. 1a). No profound rise in body temperature was observed in all groups, however significant differences in body temperature was observed in group infected with *S. aureus* (*p* ≤ 0.001; group 3) and group implanted with CPB and inoculated with *S. aureus* (*p* ≤ 0.01; group 4) on 7 and 14 dpi as compared to 0 dpi (Fig. 1b).

### Hematological analysis

**Fig. 2:**
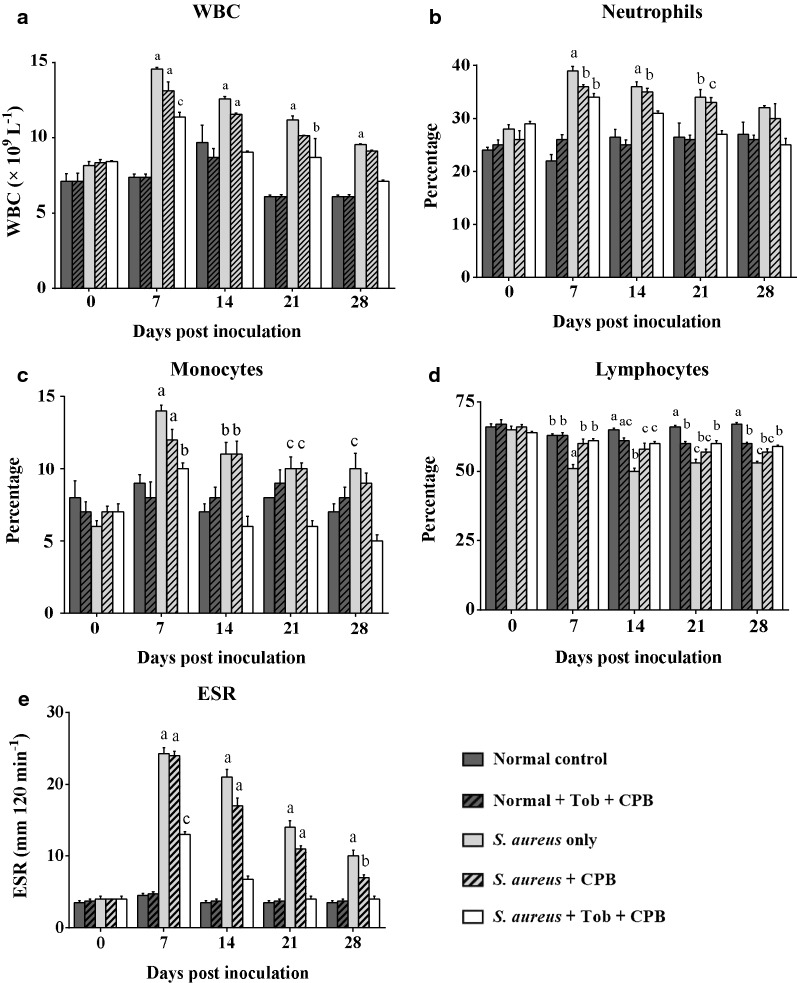
**a** Pre- and post-operative White Blood Cell concentrations for groups 1, 2, 3, 4 and 5. WBC was very high at day 7 in groups 3, 4 and 5 indicating occurrence of infection in these groups with significant difference as compared to day 7 between uninfected (1 and 2) and *S. aureus* infected (3, 4 and 5) groups. **b** Pre- and post-operative neutrophil percentages in experimental animals showing increased neutrophil counts at day 7 in *S. aureus* infected groups (3, 4 and 5) indicating the occurrence of bacterial infection in these groups (**c**) Pre- and post-operative monocyte percentages in experimental animals showing an increased monocyte counts at day 7 in *S. aureus* infected groups (3, 4 and 5). **d** Pre- and post-operative lymphocyte percentages in experimental animals showing a decrease in lymphocyte counts in *S. aureus* infected groups (3, 4 and 5) from day 7 till day 28. **e** Pre- and post-operative ESR levels in experimental animals showing a sharp increase in the *S. aureus* infected groups (3, 4 and 5) at day 7. Error bar represent the standard error of mean. ^a,b,c^Means with different superscripts are statistically significant once compared to respective day 0. Values are expressed as mean ± SEM and ^a^*p* ≤ 0.001, ^b^*p* ≤ 0.01, ^c^*p *≤ 0.05

Postoperative hematological analysis showed no significant differences in total leucocyte counts in uninfected groups. However, significant differences in total leucocyte counts were found among the infection groups, i.e., group 3 (*p* ≤ 0.001), group 4 (*p* ≤ 0.001) and group 5 (*p* ≤ 0.05) on 7 dpi as compared to 0 dpi. On 14, 21 and 28 dpi, groups 3 and 4 (*p* ≤ 0.001) showed increase in total leucocyte counts in comparison to baseline values (0 dpi) (*p* ≤ 0.001). The total leucocyte count in the infected and uninfected groups were unaltered, the total leucocyte count remained unchanged in the uninfected groups as compared to the groups having contaminated implants with/without TOB (Fig. 2a). However, based on the trend, a steady decline in the WBC count was observed in the infected groups from 7 to 28 dpi. The neutrophil counts in the infected groups (groups 3, 4 and 5) were increased at 7 dpi in comparison to the baseline values (0 dpi). Similarly, at 14 and 21 dpi, groups 3 and 4 still had higher neutrophil counts in comparison to 0 dpi (Fig. 2b). The monocyte counts were increased (*p* < 0.05) in groups 3, 4 and 5 at 7 dpi. At 14, 21 and 28 dpi, the monocyte count was higher (*p* < 0.05) in groups 3 and 4. However, a decline trend was observed from 7 dpi to 28 dpi, indicating gradual restoration of the levels to normalcy (Fig. 2c). The lymphocyte count consistently decreased (*p* < 0.05) in group 3 from 7 to 28 dpi, in comparison to counts seen at day 0 (Fig. 2d). The ESR level increased (*p* < 0.05) by three- to fivefold in the infected groups at 7 dpi. At 14 dpi, the ESR level was still higher (*p *< 0.05) in groups 3 and 4, but declined in group 5 to a level that was still higher than 0 dpi. At days 21 and 28, the ESR levels in groups 3 and 4 were still higher (*p* < 0.05) than at 0 dpi (Fig. 2e).

### Tobramycin-loaded CPB prevented experimental rabbits from developing chronic osteomyelitis

**Fig. 3:**
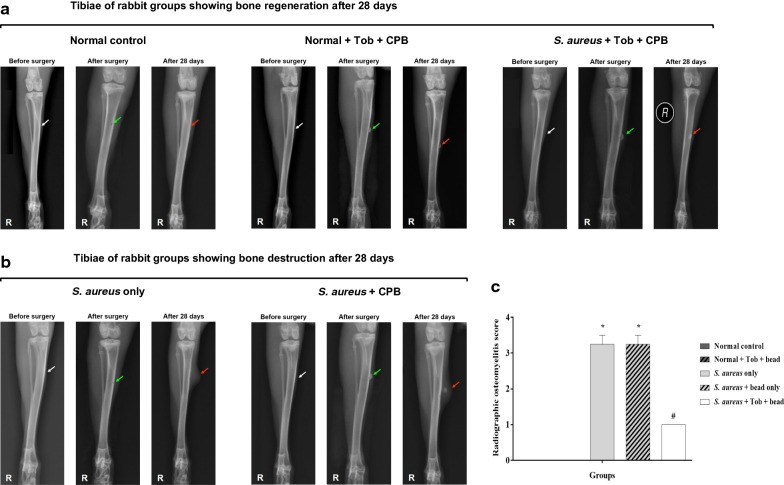
**a** Radiological examination of Normal control, Normal + TOB + CPB; red arrow indicates evident bone formation, remodelling of the bone, dissolution of the CPB and filling of the bone defect with mineral component of the CPB 28 days postoperative. Radiological examination of *S. aureus* + TOB + CPB (group 5); white arrow indicates area of the normal bone to be incised and radiological condition of the bone before incision; green arrow indicates incision point (bone defect) and implanted TOB incorporated CPB; red arrow indicates inhibition of inflammation by TOB, new bone formation, remodelling of bone, dissolution of CPB and filling of the bone defect with mineral component of CPB 28 dpi. **b** Radiological examination of *S. aureus* infected group (group 3); green arrow indicates point of incision (bone defect) and bacteria inoculation spot; red arrow indicates swelling due to inflammatory edema, abscess formation, soft tissue abnormality, inflammation, periosteal reaction, bone destruction, osteomalacia at 28 dpi (osteomyelitis formation). Radiological examination of *S. aureus* + CPB group (group 4); white arrow indicates area of the normal bone to be incised and radiological condition of the bone before incision; green arrow indicates point of incision (bone defect), point of bacterial inoculation and CPB implantation; red arrow indicates swelling due to inflammatory edema, abscess formation, soft tissue abnormality, displacement of the implanted CPB from the original position due to inflammation, periosteal reaction, bone destruction and osteomalacia at 28 dpi (osteomyelitis formation), R = right tibia. **c** Quantification of X-ray images of experimental rabbits (groups 1–5) respectively. Error bars represent the standard error of mean. *p < 0.05

Several radiological parameters corresponding to osteomyelitis infection, such as changes in bone morphology, periosteal elevation and periosteal thickening together with meta- and diaphyseal osteolysis were observed in X-ray radiographs, which facilitated the use of a modified scoring system [23] for osteomyelitis (Table 1). After 28 dpi, the control groups showed no signs of abnormal morphology of the tibia; no closure of the bone defect and no displacement of the implanted CPB (Fig. 3a). However, the test group, i.e., *S. aureus* + TOB + CPB showed mild or no periosteal reaction with closure of the defect at 28 dpi (Fig. 3a). Whereas, signs of chronic osteomyelitis with periosteal reaction, osteolysis proximal to metaphyseal level and diaphyseal osteolysis with cortical resorption were noticed in the contaminated implant groups, i.e. *S. aureus* only and *S. aureus* + CPB at 28 dpi (Fig. 3b). Assessments of the computed individual radiographs by use of the modified scoring system revealed a significantly greater score in the groups inoculated with *S. aureus* only and *S. aureus* + CPB (Fig. 3c).

### Tobramycin-loaded CPB exhibited strong antibacterial activity against *S. aureus*

**Fig. 4:**
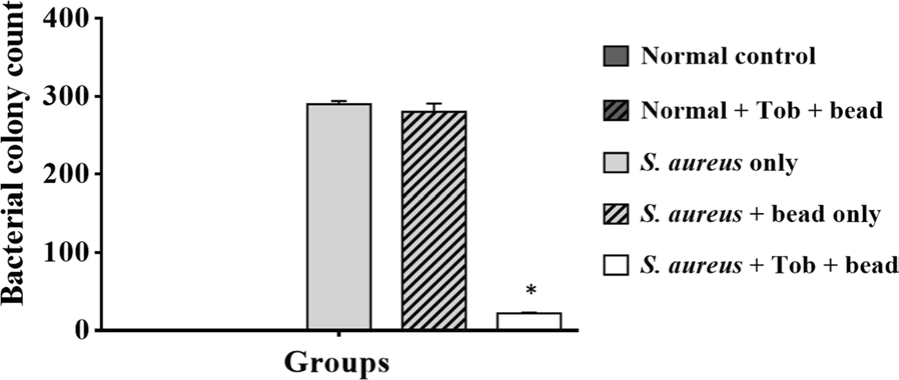
Quantitative microbiological analysis. Average counts of CFU/g bone tissue (± SEM) at day 28 are plotted. The *S. aureus* + TOB + CPB group have significantly (*p = 0.0014) reduced bacterial load compared with the control group 3 (*S. aureus* only). Group 4 (*S. aureus* + CPB) showed a slight tend towards reducing the bacterial load. Error bar represent the standard error of the mean

The quantitative bacteriological data from each group is shown in Fig. 4. Bone homogenate samples tested for the presence of *S. aureus* in the normal control and normal control + CPB groups showed the absence of *S. aureus* in these groups. Untreated groups, i.e. *S. aureus* only and *S. aureus* + CPB groups showed dense colonization of *S. aureus* growth ranging from ~ 3.8 to 4 log_10_ CFU/g of bone tissue. A slight reduction in CFUs (0.1 log_10_ reduction in CFU) was observed in *S. aureus* + CPB group compared to the control (*S. aureus* only). Similarly, the test group; *S. aureus* + TOB + CPB showed 3.2–3.4 log_10_ reduction in CFU per gram of bone tissue compared to controls (Fig. 4). Microbiological analysis revealed that TOB-loaded CPB formulation exhibited strong anti staphylococcal activity.

### Tobramycin-loaded CPB groups exhibited enhanced new bone formation and remodeling of bone defects

**Fig. 5:**
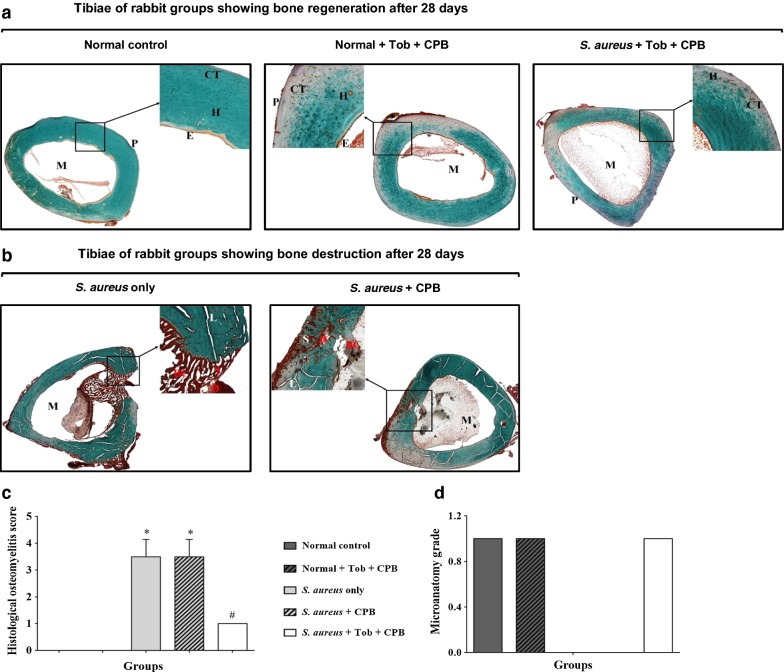
Histological images of bone sections harvested at 28 dpi and stained with Masson Goldner’s trichrome. **a** Group 1, group 2 and group 5 showing mild periosteal reaction consisting of normal bone architecture; slight distribution of immature connective tissue (CT), numerous active harversian canals (H), endosteum-layer (E) and marrow cavity. **b** Bone sections of groups 3 and group 4 showing osteomyelitis consisting of progressive destruction of bone architecture, mineral component of the bone and no bone formation at the defect site (D), note the bacterial colony (BC), empty lacunae (L) and scattered sequestra (S). ×40 magnification. **c** Quantification of histological images of experimental rabbits (groups 1–5) respectively, after 28 days follow up. Error bar represent the standard error of mean. *p < 0.05. **d** Quantification of histological images of experimental rabbits (groups 1–5) after 28 days follow-up. Confirmation of new bone formation and microanatomical changes. ×40 magnification

Tissue sections from uninfected groups showed normal bone appearance, repaired bone defect and recanalization of medullary cavity (Fig. 5a). The treatment group, i.e. *S. aureus* + TOB + CPB appeared normal with mild periosteal reaction, repaired bone defect and recanalization of the medullary cavity (Fig. 5a). However, the *S. aureus* only and *S. aureus* + CPB groups showed progressive destruction of the bone architecture and mineral component, without observable bone formation at the affected sites (Fig. 5b). The quantitative differences in osteomyelitis rating and microanatomy status between the various groups are shown in Tables 1 and 2, respectively. The scores indicated new bone formation in the uninfected controls (Normal control and Normal + TOB + CPB) and the test group (*S. aureus* + TOB + CPB), respectively (Fig. 5c, d).

## Discussion

In the present study, we used a prophylactic and a therapeutic model which was partially modified based on previously published models [14, 25]. Moreover, many of the most relevant infection parameters were combined in a single animal model and these parameters were measured on a daily basis for 28 consecutive days. To additionally ensure a valid osteomyelitis model with a full blown infection in the *S. aureus* inoculated groups, the inoculum size was slightly increased. A 100 µL inoculum of *S. aureus* containing 10^6^ CFU/mL is ideal to cause high rate of infection in the presence of carrier matrices like PMMA which is much similar to CPB [22, 25]. However, in this study 10^7^ CFU/mL was used which resulted in a full blown infection in the contaminated bead implant groups.

Antibiotic-impregnated beads, including CPB are commonly used to decrease the adverse systemic reactions and obtain local therapeutic levels of antibiotics [26]. In this study, TOB-incorporated CPB, irrespective of the relatively large inoculum (10^7^ cfu), did not result in sepsis development in the animals. Another significance of this study is the excellent placement of the CPB in the tibial cavity. The placement of antibiotic beads has been shown to affect antibiotic tissue levels in the bone [15]. Localized treatment of osteomyelitis involves insertion of antibiotic beads into the tibial cavity thus adding to the clinical significance of this model [13].

With respect to the antibacterial potency of the TOB-loaded CPB, the results indicate that 30 mg/mL of TOB incorporated CPB was sufficient in inhibiting the growth of *S. aureus* in vivo, as evidenced by the lower bacterial load in comparison to the controls (*p* = 0.0014) (Fig. 4). The rabbit group inoculated with *S. aureus* only demonstrated a trend (*p* ≤ 0.001) towards increased bacterial growth per gram of bone. This in turn suggests a dose response effectiveness of TOB in vivo (Fig. 4). On the other hand, the group implanted with TOB incorporated CPB without bacterial inoculums showed no bacterial growth in their tibia (Figs. 4, 5a). Similar findings were observed in the sham group.

The dose of TOB incorporated in the CPB (30 mg/mL) was sufficient enough to prevent infection in the experimental rabbits. In our earlier investigation on the in vitro elution and dissolution studies using TOB, incorporating TOB with CPB resulted in a slow residual release of the antibiotic from 30 min to 1344 h (8 weeks) and dissolution of calcium phosphate. Moreover, the mean TOB release in the TOB-incorporated CPB displayed a gradual increase from 10.0 µg/mL (168 h) to 60.7 µg/mL (1344 h) which suggests that the rate of drug release was exponentially related to the release time [20]. This slow and steady release of TOB from CPB can be attributed to the bactericidal action of TOB against *S. aureus* as observed in this study. Furthermore, the dose of TOB released over time resulted in an amount that was optimal in killing the bacterial load present in 1 g of bone. It is therefore difficult to relate the very high release characteristics of TOB in vitro to the results obtained in vivo. Further confirmation is needed on the steady release and rapid clearance of TOB in the animal system. An earlier investigation by Den Hertog et al. [27] showed that TOB antibiotic is susceptible to degradation by proteolytic enzymes. This could possibly reduce local tissue levels of TOB in vivo [28]. In the present study, a consolidation of these factors could have resulted in diminished local complementary or active tissue concentrations of tobramycin, feasibly compromising the in vivo efficacy. This underlines the relatively better efficacy of TOB incorporated CPB in preventing osteomyelitis.

Previous studies have shown the importance of antibiotics incorporated to cement spacers or PMMA and their elution properties. One major advantage of these implants is that antibiotics are released from the carriers in such a way that the local levels of antibiotic vastly exceed the minimum inhibitory (MIC) or bactericidal (MBC) concentrations needed to treat most pathogenic organisms, and that these levels are much higher than those achieved with parenteral therapy [29]. Such high concentrations and a steady drug release are important, especially when TOB is used for impregnation of bone cement/CPB, because this aminoglycoside has a peak dose effect for bactericidal activity [30, 31]. A steady release and detectable concentration of TOB from carrier beads for 28 days has also been reported elsewhere [32].

It should be noted that a slight reduction in CFUs (0.1 log_10_CFU reduction) was observed in the additional control i.e., group 2 (group receiving CPB with TOB). CPB is not known to possess any antibacterial activity; however, further investigation is required in order to confirm whether the slight reduction in CFUs observed in this group was exerted by CPB or due to immunological factors in the host. Besides, we only evaluated one bacterial species (*S. aureus* strain Xen 29), which means that the results obtained here may not be extrapolated in the scenario of osteomyelitis caused by clinical *S. aureus* in hospital settings. One limitation of this study is the use of mannitol salt agar only for the presumptive identification of *S. aureus*. The use of mass spectrometry MALDI-TOF for the confirmation of the presumptive *S. aureus* was not carried out in this study and would have confirmed the *S. aureus* identified. Furthermore, the use of an antibiotic resistant clinical isolate of *S. aureus* from a osteomyelitis positive patient would have had ample acerbity to steadily generate osteomyelitis in vivo which further broaden the applicability of this acute model. Thus, the data obtained here must therefore be considered only as indicative. We note that, similar in vitro observations have also been made for tobramycin alone and in combination with vancomycin [33–35], suggesting that detailed efficacy studies may be of interest.

Neutrophils are the first line of response in inflammation. In most bacterial infections, there is an accompanying leucocytosis with neutrophila [36]. Neutrophils recruited to the site of infection release chemotactic factors that attract other neutrophils and leucocytes to the site of injury. In this study, apparent leucocytosis with neutrophila was observed in all the infected groups at 7 dpi, and subsequently in the infected untreated groups at 14 and 21 dpi. However, the group treated with TOB incorporated CPB had lower total WBC and neutrophil count in comparison to the infected untreated groups; this is due to the effect of TOB which resulted in reduced bacterial population at the site of injury and thus lower systemic leucocyte response.

## Conclusions

The use of TOB-incorporated CPB at the sites of bone infection can be adopted as a standard care since CPB enabled the localization of supra-MIC levels which might have been difficult to achieve with other substitutes. Tobramycin-incorporated CPB effectively promoted soft tissue and bone healing, and prevented early bacterial colonization by *S. aureus* in infected tibia. CPB showed promising antistaphylococcal activity in treating chronic osteomyelitis when combined with TOB, with long periods of sustained efficacy. This profile is consistent with the properties required for the development of a potential antibacterial for staphylococcal osteomyelitis and supports continued investigation of this formulation. Future interests in the development of TOB as a traditional ‘drug of choice’ antibiotic will largely be focused on addressing issues related to its pharmacokinetics and inhibition of enzymatic proteolysis. Data obtained from the present study will serve as a basis for more progress in the development of TOB for the treatment of MDR pathogens in hospital settings.

## Additional file

**Additional file 1: Figure S1.** Summary of surgical procedure to induce osteomyelitis and implantation of the CPB in the rabbits.

## Abbreviations

ATCC: American Type Culture Collection
CFU: colony forming units
CPB: calcium phosphate beads
dpi: days postinfection
ESR: erythrocyte sedimentation rate
TSA: trypticase soy agar
MDR: multidrug resistant
PMMA: poly(methyl methacrylate)
TOB: tobramycin
WBC: white blood cells
