# Supplementary figures and images for: In vivo efficacy of tobramycin-loaded synthetic calcium phosphate beads in a rabbit model of staphylococcal osteomyelitis
Source: Ann Clin Microbiol Antimicrob. 2018 Dec 28;17:46. doi: 10.1186/s12941-018-0296-3 (PMC6309062; doi:10.1186/s12941-018-0296-3)

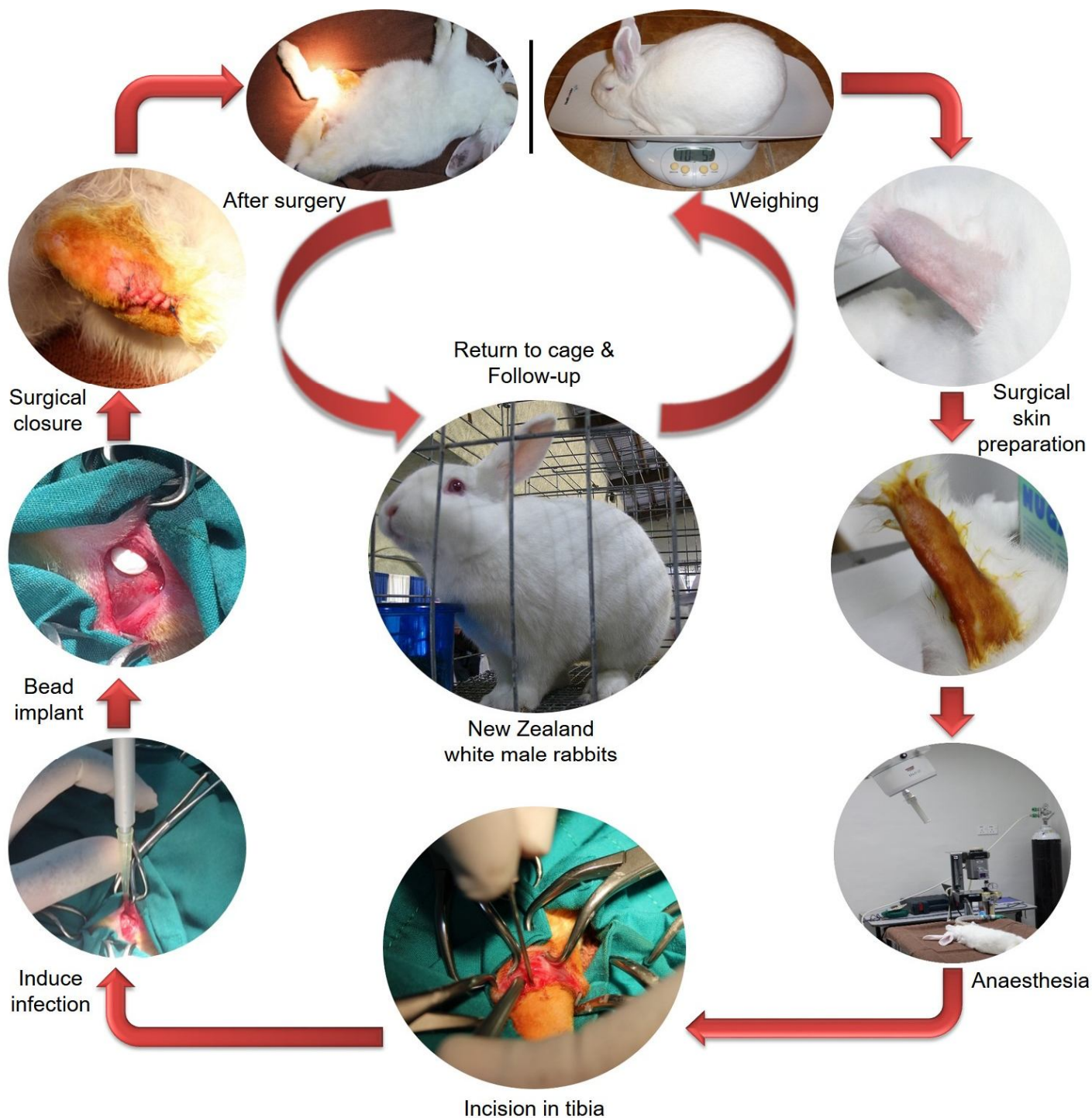

Supplement: Supplementary file 1 — Additional file 1: Figure S1. Summary of surgical procedure to induce osteomyelitis and implantation of the CPB in the rabbits. [file 12941_2018_296_MOESM1_ESM.pdf]
